# INTERACTIVE VOICE ASSISTANT ROUTINES: A USABILITY STUDY

**DOI:** 10.1093/geroni/igad104.2819

**Published:** 2023-12-21

**Authors:** Marcia Shade, Changmin Yan, Julie Blaskewicz Boron, Valerie Jones

**Affiliations:** University of Nebraska Medical Center, Omaha, Nebraska, United States; UNL, Lincoln, Nebraska, United States; UNO, Omaha, Nebraska, United States; UNL, Lincoln, Nebraska, United States

## Abstract

Musculoskeletal pain is prevalent among older adults and is associated with disability and social isolation. This study explored the feasibility of using conversational voice assistant (CVA) routines for proactive interaction and pain reduction with older adults. A 12-week randomized controlled pilot trial was conducted with adults aged 60 years and older who self-reported living alone and chronic musculoskeletal pain. Participants (N=37) were randomly assigned to a standard routine or an enhanced routine group. Data were collected on participants’ CVA engagement, self-reported pain, and system usability. Overall, older adults-initiated morning routines more frequently than the evening routines. The enhanced group initiated more morning routines (M=50.01; SD=33.07) than the standard group (M=16.11, Z= -1.70, p<.05, r= -.28). Participants reported significant post-study pain reduction, t(35)=1.84, p<.05, r= .30 (baseline mean=3.60, SD=1.93; post-study mean=3.19, SD=1.97); there were no significant group differences in amount of pain reduction. The enhanced group participants rated the system usability as good (Mean=74.81, SD=9.28); those in the standard condition reported a lower-tier rating of “OK” (Mean=65.88, SD=12.76), t(31)= -2.29, p<.05, r=.37. System usability predicted pain reduction (B= -0.55, t(13)= -2.46, p<.05) in the enhanced condition (R2=.30, F(1,14)=6.07, p<.05), but the association was non-significant in the standard condition (R2=.14, F(1,15)=0.29, p=.60). These results show that the CVA routines were feasible to use with older adults, and resulted in perceived pain reduction. Future creation of tailored routines based on the older adults’ pain characteristics, personality, and interaction preferences when alone, may further increase engagement.

